# Anti-inflammatory effects of dexmedetomidine on human amnion-derived WISH cells

**DOI:** 10.7150/ijms.49909

**Published:** 2020-09-09

**Authors:** Sang-Hun Shin, Jae-Chaul You, Ji-Hye Ahn, Yeon Ha Kim, Ji-Uk Yoon, Ah-Reum Cho, Eun-Jung Kim

**Affiliations:** 1 Department of Oral and Maxillofacial Surgery, School of Dentistry, Pusan National University, Yangsan, Korea.; 2 Department of Dental Anesthesia and Pain Medicine, School of Dentistry, Pusan National University, Dental Research Institute, Yangsan, Korea.; 3 Department of Integrated Biological Science, Pusan National University, Busan 46241, Korea.; 4 Department of Anesthesia and Pain Medicine, School of Medicine, Pusan National University, Yangsan, Korea.; 5 Department of Anesthesia and Pain Medicine, Pusan National University, School of Medicine, Yangsan, Republic of Korea.

**Keywords:** Amnion, Dexmedetomidine, Inflammation, p38, NF-κB

## Abstract

**Background:** To maintain the normal pregnancy, suppression of inflammatory signaling pathway is a crucial physiologic response. Dexmedetomidine has been used for labor analgesia or supplement of inadequate regional analgesia during delivery. And it has been reported that dexmedetomidine has an anti-inflammatory effect. In this study, we examined the influence of dexmedetomidine on the expression of cyclooxygenase-2 (COX-2), prostaglandin E_2_ (PGE_2_) and inflammatory cytokines in lipopolysaccharide (LPS)-stimulated human amnion-derived WISH cells. In addition, we evaluated the association of mitogen-activated protein kinase (MAPK) and nuclear factor kappa B (NF-κB) pathway in anti-inflammatory effect of dexmedetomidine.

**Methods:** Human amnion-derived WISH cells were pretreated with various concentrations of dexmedetomidine (0.001-1 µg/ml) for 1 h and after then treated with LPS (1 µg/ml) for 24 h. MTT assay was conducted to evaluate the cytotoxicity. Nitric oxide (NO) production was analyzed using Griess-reaction microassay. RT-PCR was performed for analysis of mRNA expressions of COX-2, PGE_2_, tumor necrosis factor (TNF)-α and interlukin (IL)-1β. Protein expressions of COX-2, PGE_2_, p38 and NF-κB were analyzed by western blotting.

**Results:** LPS and dexmedetomidine had no cytotoxic effect on WISH cells. There was no difference in NO production after dexmedetomidine pretreatment. The mRNA and protein expressions of COX-2 and PGE_2_ were decreased by dexmedetomidine pretreatment in LPS-treated WISH cells. Dexmedetomidine also attenuated the LPS-induced mRNA expression of TNF-α and IL-1β. The activation of p38 and NF-κB was suppressed by dexmedetomidine pretreatment in LPS-treated WISH cells.

**Conclusion:** We demonstrated that dexmedetomidine pretreatment suppressed the expressions of inflammatory mediators increased by LPS. In addition, this study suggests that anti-inflammatory effect of dexmedetomidine on WISH cells was mediated by the inhibitions of p38 and NF-κB activation.

## Introduction

The endogenous levels of prostaglandins (PGs) in the endometrial decidua of pregnant women are 200-fold lower than that of non-pregnant women 1. The selective epigenetic silencing of the key inflammatory chemokine genes that attract T cells in the decidual stroma is maintained during normal pregnancy 2. The suppression of inflammatory signaling pathways is critical for maintaining normal pregnancy. The levels of PGs in the plasma, urine, and amniotic fluid increase before the onset of labor leading to the rupture of the fetal membrane, cervical dilatation, and myometrial contractions 3, 4. The administration of exogenous PGs or the failure to inhibit PG production can result in spontaneous abortion 5. Besides PGs, nitric oxide (NO) is one of the factors involved in the maintenance of normal pregnancy. NO is a short-lived free radical generated from L-arginine and preserves the uterine relaxation during pregnancy 6, 7. Although NO is known as a relaxing factor in the myometrium, it is not clear how NO production is altered by an inflammatory response.

Inflammation has been recognized as a defensive immune response against pathogens. During intrauterine infections, innate immune responses activate inflammatory reactions leading to the secretion of pro-inflammatory cytokines that initiate the signaling pathway leading to preterm labor 8. The inflammatory reaction is enhanced by various molecules. Reactive oxygen species (ROS) is a molecule involved in the progression of inflammatory responses. Antioxidant molecules can reduce oxidative stress by ROS and suppress inflammatory responses 9. Bacterial components such as lipopolysaccharide (LPS) and lipoteichoic acids are pathogen-associated molecules that trigger inflammatory responses 10, 11.

LPS is the most abundant component of the outer membrane of gram-negative bacteria. It is a key stimulator of acute inflammatory responses and triggers the release of various pro-inflammatory cytokines including tumor necrosis factor-alpha (TNF-α), interleukin (IL)-1β, and IL-6 12-14. Toll-like receptor 4 (TLR4) is an innate LPS-pattern recognition receptor that is important for a rapid immune response and proper bacterial clearance 15. During LPS-induced inflammatory response, the binding of LPS to TLR4 leads to the activation of the mitogen-activated protein kinase (MAPK) and nuclear factor kappa B (NF-κB) signaling pathways 16,19. LPS also enhances the production of the cyclooxygenase‑2 (COX-2) that is essential for the production of PGs 18. Based on this evidence, we treated WISH cells with LPS to induce an inflammatory response and expected that activation of MAPK and NF-κB may be affected by the LPS-induced inflammatory process.

Dexmedetomidine is a highly selective α-2 adrenoreceptor agonist that has been used for achieving sedation, anxiolysis, and analgesia in intensive care units (ICUs) or during operative procedures. Compared to other sedative drugs, dexmedetomidine has a low risk of respiratory depression 19,20. Consequently, the use of dexmedetomidine in ICUs and procedural sedation has increased. Previous studies reported that dexmedetomidine has anti-inflammatory effects; dexmedetomidine was shown to decrease the secretion of inflammatory cytokines and consequently suppress inflammatory responses in animal experiments and clinical studies 21,22. Dexmedetomidine can be administered to pregnant women for labor analgesia when the patient refuses epidural or spinal anesthesia or as a supplement to inadequate epidural analgesia. However, physicians are reluctant to use dexmedetomidine in pregnancy because of the possibility of uteroplacental transfer and the effects on the fetus 23.

This study was conducted to investigate the effect of dexmedetomidine on the expression of inflammatory mediators in LPS-stimulated human amnion-derived WISH cells. The involvement of the MAPK and NF-κB signaling pathways in the anti-inflammatory effect of dexmedetomidine was examined.

## Materials and Methods

### Cell culture

The WISH human amnion cell line was purchased from the American Type Culture Collection (ATCC; Manassas, VA, USA) and cultured in Eagle's Minimum Essential Medium (ATCC) supplemented with 10% fetal bovine serum (Gibco, Carlsbad, CA, USA) at 37 °C in an atmosphere of 5% CO_2_. The WISH culture was subcultured (passaged) once every 3 days.

### Dexmedetomidine treatment

A commercially available formulation of dexmedetomidine hydrochloride (KyongBo Pharm, Chungnam, Korea) was used in this study. The formulation was diluted with the culture medium and added to the cultures at concentrations of 0.001, 0.01, 0.1, and 1 μg/mL and incubated for 1 h followed by treatment with the growth medium or 1 μg/mL LPS (Sigma, St. Louis, MO, USA) for 24 h.

### Nitric oxide (NO) assay

To evaluate the effect of pretreatment with dexmedetomidine on the production of NO in LPS-treated WISH cells, the Griess reaction microassay (Cell Signaling Technology) was performed. WISH cells were seeded on 24-well plates at a density of 1 × 10^4^ cells/well. The concentration of NO in the culture supernatant was estimated from the concentration of nitrites (NO_2_^-^) that are stable end-products of NO metabolism. WISH cells were pretreated with dexmedetomidine at concentrations ranging between 0.01 and 1 μg/mL for 1 h and subsequently incubated with 1 μg/mL LPS for 24 h. Following incubation, the supernatants were collected and mixed with an equal volume (1:1) of Griess reagent. The samples were incubated at room temperature for 10 min and their absorbance was measured at 540 nm using a microplate reader (Bio-Rad Model 680; Bio-Rad, Hercules, CA, USA).

### MTT assay

WISH cells were seeded in 24-well plates at a density of 1 × 10^5^ cells/well and cultured for 24 h at 37 °C in an incubator with an atmosphere of 5% CO_2_. The cells were subsequently exposed to dexmedetomidine at concentrations ranging between 0.001 and 1 μg/mL for 3 h. Following dexmedetomidine treatment, the MTT [3-(4,5-dimethylthiazol- 2-yl) -2,5-diphenyl tetrazolium bromide; Affymetric, Inc. USB, OH, USA] assay was performed by adding 100 μL MTT solution (5 mg/mL in PBS at pH 7.4) to each well and incubating at 37 °C. The medium was removed after 1 h and 100 μL dimethyl sulfoxide (DMSO; Biosesang, Seongnam, Korea) was added to each well. The plate was gently rotated on an orbital shaker for 15 min to dissolve the precipitate. The absorbance was measured at 540 nm with a microplate reader (Bio-Rad Model 680). All the experiments were performed at least three times.

### RNA isolation and reverse transcriptase-polymerase chain reaction (RT-PCR)

WISH cells were seeded in a 12-well cell-culture plate at a density of 5 × 10^5^ cells/well. After 1 h of treatment with dexmedetomidine, the cells were treated with LPS for 24 h. Following LPS treatment, the total RNA was isolated using 500 μL of TRIzol reagent (Invitrogen, Carlsbad, CA, USA). The total mRNA (1 μg) was used as the template for cDNA synthesis using oligo(dT) PrimeScript^TM^ 1st strand cDNA Synthesis Kits (TaKaRa Clontech, BD Biosciences, Palo Alto, CA, USA) according to the manufacturer's instructions. RT-PCR was performed on a SimpliAmp Thermal Cycler (Applied Biosystems, Life Science Technologies, CA, USA). The primers used for PCR were: IL-1β, Forward: 5′-CTCGCCAGTGAAATGATGGCT-3′, Reverse: 5′-GTCGGAGATTCGTAGCTGGAT-3′; TNF-α, Forward: 5′-CCAGGCAGTCAGATCATCTTC-3′, Reverse: 5′-GTTATCTCTCAGCTCCACGC-3′; β-actin, Forward: 5′-GACCTGACTGACTACCTCATG-3′, Reverse: 5′-CGCTCATTGCCAATGGTGATG-3′. IL-1β/β-actin was amplified using 35 cycles of PCR at 95 °C for 30 s, 55 °C for 30 s, and 72 °C for 30 s, and the final extension was performed at 72 °C for 7 min. TNF-α/β-actin was amplified using 35 cycles of PCR at 94 °C for 30 s, 54 °C for 30 s, and 72 °C for 30 s, with the final extension performed at 72 °C for 10 min. The PCR products were separated on a 1.5% stained agarose gel. They were assayed using a Gel Doc ImageQuant LAS 500 System (GE Healthcare Bio-Sciences AB, Uppsala, Sweden). β-actin was used for normalizing all the target genes. The data were analyzed using ImageJ software (National Institutes of Health, Bethesda, MD, USA).

### Western blotting

All the cells were extracted with chilled RIPA buffer (50 mM Tris at pH 7.5, 150 mM NaCl, 5 mM EDTA, 0.5% NP40, 5 mM DTT, 0.2 mM sodium orthovanadate, 100 mM NaF, and 1 mM PMSF) containing 1X protease inhibitor/phosphatase inhibitor cocktail (Cell Signaling Technology). The samples (25 μg protein/well) were separated by sodium dodecyl sulfate-polyacrylamide gel electrophoresis and transferred to nitrocellulose membranes (GE Healthcare, Chicago, IL, USA). The membranes were blocked with TBS-0.1% Tween-20 (TBST) containing 3% skim milk for 1 h. The membranes were subsequently incubated overnight with α-tubulin (1:1000; Santa Cruz, CA, USA), p38 MAPK (1:1000; Cell Signaling Technology), phospho-p38 MAP kinase (1:500; Cell Signaling Technology), NF-kB p65 (1:1000; Santa Cruz), phospho-NF-kB p65 27. Ser 536 (1:500; Santa Cruz), PGE synthase 2 (A-2) (1:1000; Cell Signaling Technology), and COX-2 (D5H5) rabbit mAb (1:1000; Santa Cruz) antibodies at 4 °C in TBST with 3% skim milk. After washing three times with TBST, the membranes were incubated with horseradish peroxidase-conjugated anti-rabbit (1:1000; Enzo Life Sciences, Plymouth Meeting, PA, USA) and anti-mouse (1:1000; Santa Cruz) antibodies for 1 h at room temperature. The membranes were subsequently washed three times with TBST and the bands were visualized using ECL detection reagents (Promega, Madison, WI, USA). The expression of α-tubulin was used as a control. The target-protein bands were normalized relative to the control band with ImageJ software.

### Statistical analyses

Data are presented as the mean ± standard deviation (SD). All the experiments were repeated at least three times. The statistical analyses were performed using the SigmaPlot v10 software. *P* value of < 0.05 was considered to be statistically significant.

## Results

### Cytotoxicity of LPS and dexmedetomidine pretreatment in WISH cells

**Figure 1:**
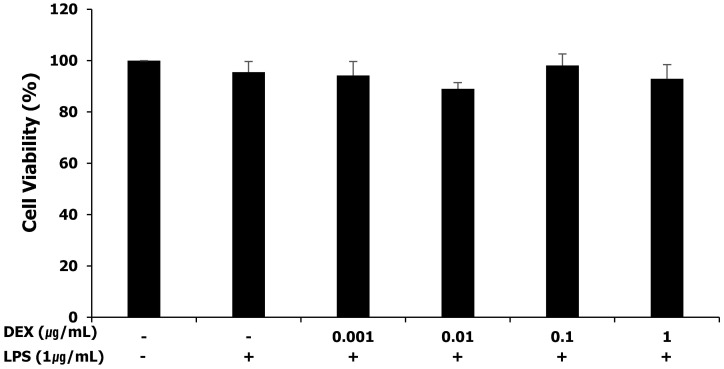
Effect of LPS and dexmedetomidine pretreatment on cytotoxicity in WISH cells was measured by MTT assay. Data are presented as mean ± SD. All experiments were repeated three times. DEX: dexmedetomidine.

The MTT assay was performed for assessing the cytotoxicity of LPS and dexmedetomidine pretreatment in WISH cells. WISH cells were treated with concentrations of dexmedetomidine ranging between 0.001 and 1 μg/mL followed by treatment with 1 μg/mL LPS. As shown in **Figure 1**, LPS and dexmedetomidine pretreatment did not affect the viability of WISH cells indicating that LPS and dexmedetomidine were not cytotoxic to WISH cells.

### NO production in LPS-treated WISH cells after dexmedetomidine pretreatment

**Figure 2:**
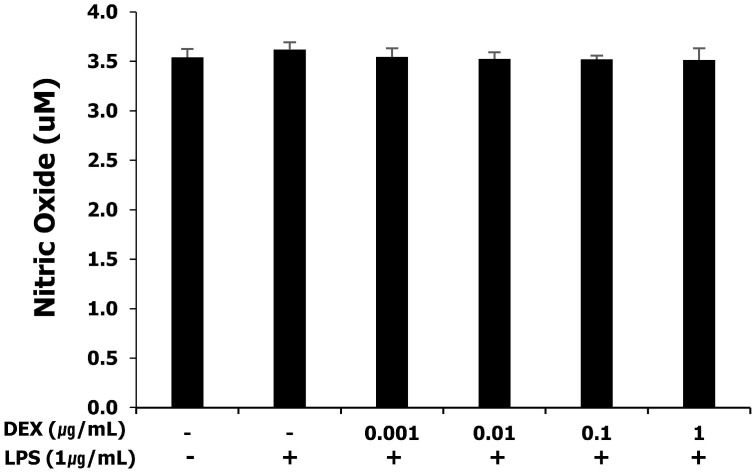
Effect of dexmedetomidine pretreatment on NO production in LPS-treated WISH cells was investigated by the Griess-reaction microassay. Data are presented as mean ± SD. All experiments were repeated three times. DEX: dexmedetomidine.

NO production was measured as nitrites by the Griess-reaction microassay. WISH cells basally released nitrites into the culture medium. The release of nitrites was not affected by LPS and pretreatment with dexmedetomidine at all concentrations (**Figure 2**).

### Effect of dexmedetomidine pretreatment on COX-2 and PGE_2_ expression following LPS-induced inflammatory response in WISH cells

**Figure 3:**
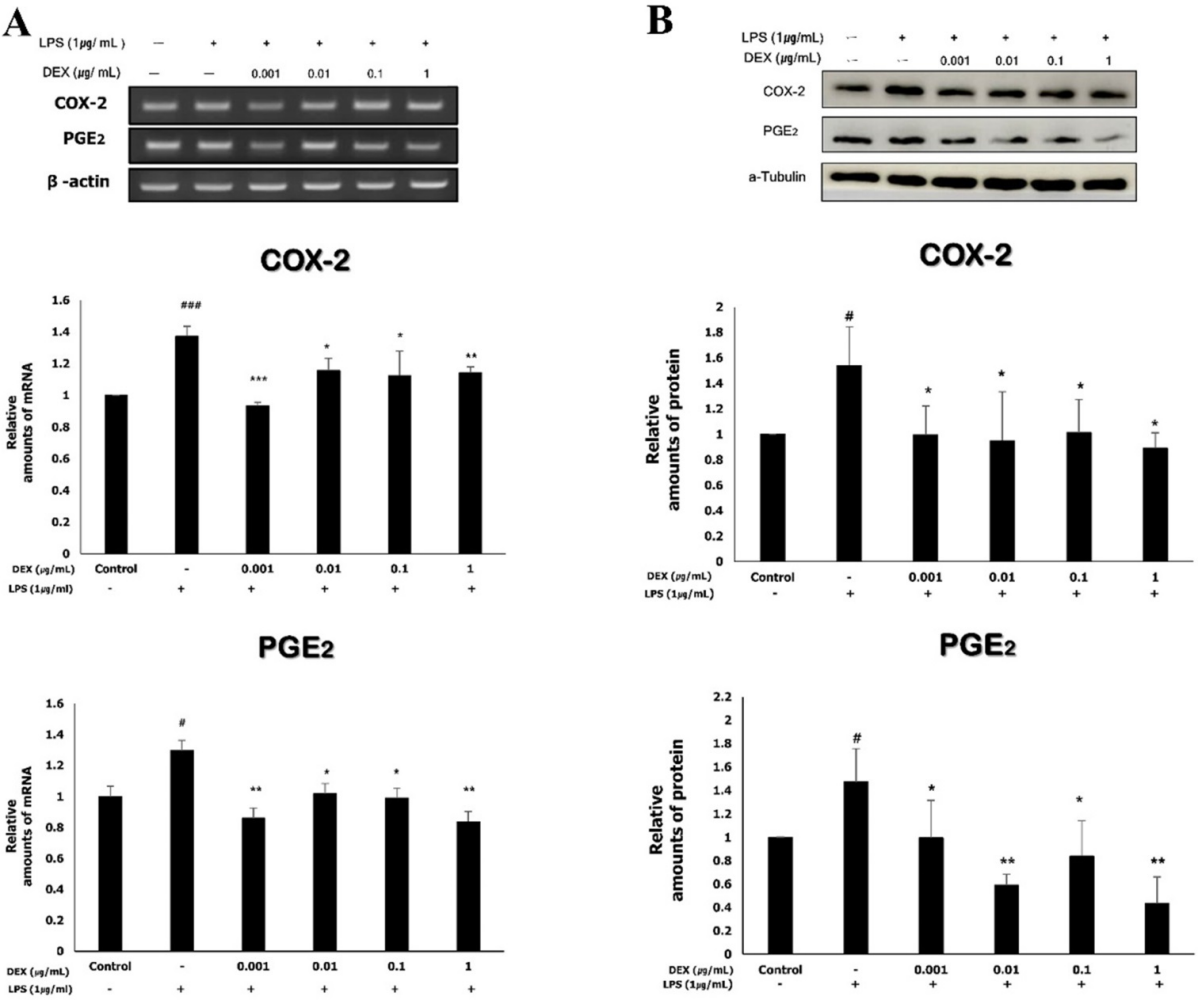
To evaluate the effects of dexmedetomidine pretreatment on the expressions of COX-2 and PGE_2_, WISH cells were pretreated with dexmedetomidine (0.001-1 µg/mL) and after then treated with 1 µg/ml LPS. (**A**) The mRNA expressions of COX-2 and PGE_2_ were determined using RT-PCR analysis. Relative density of the mRNA expression of COX-2 and PGE_2_ was normalized by β-actin. (**B**) The protein expressions of COX-2 and PGE_2_ were evaluated by western blotting. Relative density analysis was performed using ImageJ software and normalized by α-tubulin. Data are mean ± SD of three independent experiments. ^#^*P<*0.05, ^###^*P<*0.001 versus control group; *P<0.05, **P<0.01, ***P<0.001 versus LPS group. DEX: dexmedetomidine.

The mRNA expression levels of COX-2 and PGE_2_ were determined using RT-PCR. The mRNA expression of COX-2 in WISH cells significantly increased to 1.37 ± 0.06 following exposure to 1 μg/mL LPS compared to that of the control (*p<*0.001). The increased mRNA expression of COX-2 was significantly reduced by dexmedetomidine pretreatments at all concentrations (0.93 ± 0.02 at 0.001 μg/mL, *p<*0.001; 1.15 ± 0.08 at 0.01 μg/mL, *p<*0.05; 1.25 ± 0.12 at 0.1 μg/mL, *p<*0.05; 1.14 ± 0.04 at 1 μg/mL, *p<*0.01). The mRNA expression of PGE_2_ significantly increased to 1.3 ± 0.13 following exposure to 1 μg/mL LPS compared to the control (*p<*0.05). The pretreatment of LPS-treated WISH cells with dexmedetomidine significantly reduced the mRNA expression levels of PGE_2_ at all concentrations (0.85 ± 0.03 at 0.001 μg/mL, *p<*0.01; 1.02 ± 0.09 at 0.01 μg/mL, *p<*0.05; 0.99 ± 0.06 at 0.1 μg/mL, *p<*0.05; 0.84 ± 0.07 at 1 μg/mL, *p<*0.01) compared to the LPS-treated group (**Figure 3A**).

The protein expression of COX-2 and PGE_2_ was analyzed by western blotting (**Figure 3B**); the results were in concordance with those obtained for the mRNA expression of COX-2 and PGE_2_. There was a significant increase in the protein expression of COX-2 (1.54 ± 0.31) and PGE_2_ (1.47 ± 0.28) following treatment with 1 μg/mL LPS compared to the control (*p<*0.05). The protein expression of COX-2 decreased significantly when the cells were pretreated with dexmedetomidine (0.10 ± 0.23 at 0.001 μg/mL; 0.95 ± 0.38 at 0.01 μg/mL; 1.01 ± 0.26 at 0.1 μg/mL; 0.89 ± 0.12 at 1 μg/mL, *p<*0.05), compared to the LPS-treated group. The protein expression of PGE_2_ also decreased by dexmedetomidine pretreatment at all concentrations and the differences compared to the LPS-treated group were statistically significant (0.99 ± 0.32 at 0.001 μg/mL, *p<*0.05; 0.59 ± 0.10 at 0.01 μg/mL, *p<*0.01; 0.84 ± 0.30 at 0.1 μg/mL, *p<*0.05; 0.44 ± 0.22 at 1 μg/mL, *p<*0.01).

### Expression of inflammatory cytokines (TNF-α and IL-1β) in LPS-treated WISH cells following dexmedetomidine pretreatment

**Figure 4:**
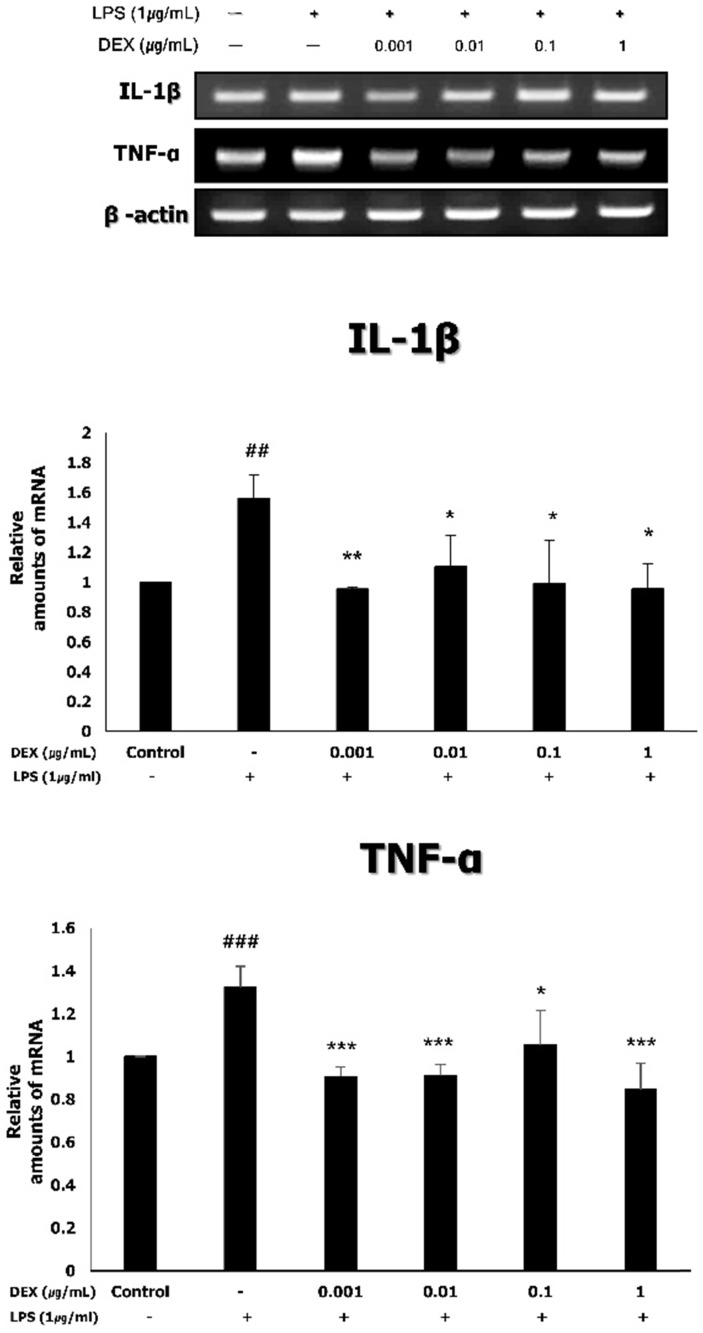
The mRNA expressions of TNF-α and IL-1β by dexmedetomidine pretreatment in LPS-treated WISH cells were measured using RT-PCR. Relative mRNA level was normalized by β-actin and presented as mean ± SD of three independent experiments. ^##^*P<*0.01, ^###^*P<*0.001 versus control group; *P<0.05, **P<0.01, ***P<0.001 versus LPS group. DEX: dexmedetomidine.

To investigate the effect of dexmedetomidine pretreatment on the expression of inflammatory cytokines, the mRNA expression of TNF-α and IL-1β was measured using RT-PCR. As shown in **Figure 4**, the treatment of WISH cells with 1 μg/mL LPS significantly increased the mRNA expression levels of TNF-α (1.33 ± 0.10, *p<*0.001) and IL-1β (1.56 ± 0.16, *p<*0.01), compared to the control. Pretreatment with dexmedetomidine significantly decreased the LPS-induced mRNA expression of TNF-α (0.91 ± 0.05 at 0.001 μg/mL, *p<*0.001; 0.91 ± 0.05 at 0.01 μg/mL, *p<*0.001; 1.05 ± 0.16 at 0.1 μg/mL, *p<*0.05; 0.85 ± 0.12 at 1 μg/mL, *p<*0.001) and IL-1β (0.95 ± 0.02 at 0.001 μg/mL, *p<*0.01; 1.10 ± 0.21 at 0.01 μg/mL; 0.97 ± 0.26 at 0.1 μg/mL; 0.96 ± 0.17 at 1 μg/mL, *p<*0.05) at all concentrations.

### Role of p38 and NF-κB in the anti-inflammatory effect of dexmedetomidine on LPS-treated WISH cells

**Figure 5:**
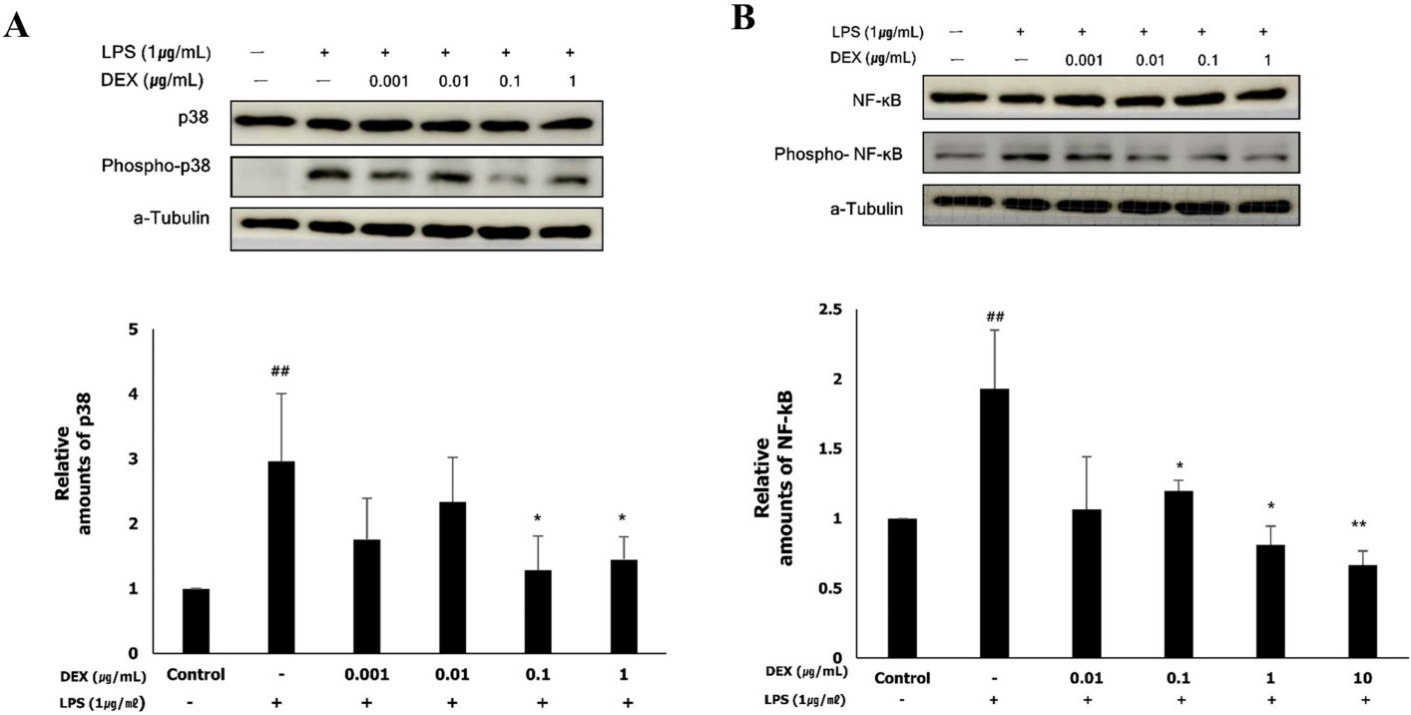
The activation of p38 and NF-κB by dexmedetomidine pretreatment in LPS-treated WISH cells were evaluated using western blotting. To investigate the activation of p38 and NF-κB, the protein expressions of phospho-p38 and phospho-NF-κB were measured. (**A**) Relative amounts of phospho-p38 protein were analyzed by triplicate experiments and normalized by p38. (**B**) Relative amounts of phospho-NF-κB protein were analyzed by triplicate experiments and normalized by NF-κB. ^##^*P<*0.01 versus control group; *P<0.05, **P<0.01 versus LPS group. DEX: dexmedetomidine.

To determine whether the anti-inflammatory effect of dexmedetomidine was mediated via the MAPK or NF-κB signaling pathways, we examined the activation of p38 and NF-κB by western blotting. LPS treatment significantly increased the protein expression of phospho-p38 (2.97 ± 0.99, *p<*0.01) and phospho-NF-κB (1.94 ± 0.43, *p<*0.01), the activated forms of p38 and NF-κB, respectively, compared to the control (**Figure 5A and B**). The protein expression of phospho-p38 was significantly decreased following pretreatment with 0.1 μg/mL (1.32 ± 0.61, *p<*0.01) and 1 μg/mL (1.51 ± 0.34, *p<*0.01) dexmedetomidine compared to the LPS-treated group (**Figure 5A**). As shown in **Figure 5B**, pretreatment with dexmedetomidine at concentrations of 0.01 μg/mL (1.19 ± 0.08, *p<*0.05), 0.1 μg/mL (0.78 ± 0.25, *p<*0.05), and 1 μg/mL (0.67 ± 0.13, *p<*0.01) significantly decreased the LPS-induced expression of phospho-NF-κB. These results suggested that pretreatment with dexmedetomidine suppresses the activation of p38 and NF-κB.

## Discussion

The present study demonstrated that pretreatment with dexmedetomidine attenuates the LPS-induced expression of COX-2, PGE_2_, and inflammatory cytokines in human amnion-derived WISH cells. To the best of our knowledge, this is the first study to investigate the effect of dexmedetomidine on the inflammatory response using WISH cells. The WISH epithelial cells are a good *in vitro* model to study the physiological functions of cells in the amnion. At the onset of labor, the levels of pro-inflammatory cytokines and chemokines increase in the epithelial cells of the amnion 24. WISH cells are the ideal candidate for *in vitro* studies for investigating the factors that trigger labor and the modulation of PGE_2_ release by various agonists 25, 26.

In this study, pretreatment with dexmedetomidine was found to have an anti-inflammatory effect on LPS-stimulated WISH cells suggesting that dexmedetomidine could antagonize the uterine contraction resulting from inflammation. Some studies have explored the effect of dexmedetomidine on inflammatory responses. Kang and coworkers 27 reported that the administration of dexmedetomidine during laparoscopic cholecystectomy decreases the secretion of inflammatory cytokines, post-operative leukocyte counts, and the levels of C-reactive protein. Studies by Taniguchi and coworkers 28 and Qiao and coworkers 29 demonstrated that dexmedetomidine reduces the concentrations of TNF-α and IL-6, and ameliorates mortality in rats with endotoxin or cecal ligation and intestinal puncture-induced sepsis. However, another study reported that dexmedetomidine increases uterine contraction at a plasma concentration of 1 × 10^-9^ g/mL 30. Karaman and coworkers 31 also demonstrated that dexmedetomidine increases the amplitude, frequency, and area under the curve of rat myometrial contractions in a dose-dependent manner. It is therefore difficult to be certain that dexmedetomidine suppresses uterine contraction by inhibiting the inflammatory response. However, it is known that dexmedetomidine has anti-inflammatory effects on WISH cells. Further studies are necessary for verifying the effect of dexmedetomidine on uterine contraction.

We treated WISH cells with LPS, a component of the cell wall of gram-negative bacteria, to induce the inflammatory response. LPS stimulates the innate immune response and is consequently recognized by LPS-binding protein (LBP) in the serum. The binding of LBP with LPS promotes the transfer to CD14 which separates the LPS and presents it to the TLR4 complex 32,33. TLRs are a family of innate immune receptors that serve as major sensors of bacterial pathogens in murine and human placenta 34-36. TLR4 is the primary receptor for transducing the LPS signal in human amniotic epithelial cells 37. Following the recognition of LPS by TLR4 in the amnion, the downstream signaling cascade is activated and leads to the activation of MAPK and NF-κB, and the induction of pro-inflammatory cytokine gene expression 38,39.

The p38 MAPKs play a major role during inflammatory responses and are rapidly phosphorylated following LPS stimulation. The p38 pathway is a key regulator of the biosynthesis of pro-inflammatory cytokines including IL-1β and TNF-α 40,41. p38α is the main isoform of p38 MAPKs and partially regulates the production of COX-2 42. NF-κB is a principal transcriptional factor involved in inflammatory responses and is translocated from the cytoplasm to the nucleus following activation. In the nucleus, the activated NF-κB binds to the promoter regions of COX-2, IL-1β, and TNF-α genes, and induces their expression 43,44. It has been established that the expression of the COX-2 gene depends on NF-κB and in turn accelerates the production of PGs that induce cervical ripening and the onset of labor 45. In our experiments, pretreatment with dexmedetomidine suppressed the LPS-induced expression of phospho-p38 and phospho-NF-κB mRNA. The LPS-induced expression of COX-2, PGE_2_, and inflammatory cytokines (IL-1β and TNF-α) was attenuated by pretreatment with dexmedetomidine. Therefore, the results of this study imply that pretreatment with dexmedetomidine restricts the LPS-induced expression of COX-2, PGE_2_, IL-1β, and TNF-α by suppressing the activation of p38 MAPK and NF-kB.

In the present *in vitro* study, WISH cells were treated with dexmedetomidine at concentrations ranging between 0.001 and 1 μg/mL (0.005 to 5 μM), based on a previous study 46. In that study, the cells were treated with dexmedetomidine at concentrations of 0.0001, 0.001, 0.01, 0.1, and 1 μg/mL. Reportedly the plasma concentrations of dexmedetomidine in clinical use ranges from 0.49 to 8 ng/mL 47. Ebert and coworkers reported that the plasma concentrations of dexmedetomidine for inducing a mild to moderate sedative effect range between 0.7 and 1.2 ng/mL. Deep sedation is thought to arise at plasma concentrations above 1.9 ng/mL 48,48. Another study reported that 0.1, 1, and 10 μM of dexmedetomidine are high concentrations for clinical use, and 0.001 μM of dexmedetomidine is the lowest concentration for clinical use 50. Therefore, the concentration of dexmedetomidine used in this study fall within the concentration range used in the clinics.

Although the effects of dexmedetomidine on the expression of inflammatory substances in LPS-stimulated WISH cells were evaluated under normoxic conditions, the inflammation is often accompanied by hypoxia owing to the high oxygen consumption 51. Therefore, it is uncertain whether our experiments were conducted under normoxia or hypoxia. The role of hypoxia in the inflammatory response is still unclear. To demonstrate the effect of hypoxia on the inflammatory response in the amnion, further research is needed.

In this study, we used human amnion-derived WISH cells as a model for studying the effect of dexmedetomidine on the expression of inflammatory substances involved in preterm labor. In previous studies, various animal models and human-tissue models were used to examine the effect of inflammation on preterm labor. Despite significant differences in reproductive biology to humans, animal models such as nonhuman primates, sheep, and mice have been useful to study preterm labor 52-54. Some studies used human amnion epithelial cells separated from the term placenta delivered by elective cesarean section to analyze the inflammatory effects of various agents on amnion 55,56.

In conclusion, pretreatment of WISH cells with dexmedetomidine attenuated the LPS-induced expression of inflammatory substances including COX-2, PGE_2_, TNF-α, and IL-1β. This anti-inflammatory effect of dexmedetomidine suggests that dexmedetomidine may help reduce the uterine contractions caused by the inflammatory response during pregnancy. The present *in vitro* study further demonstrated that dexmedetomidine mediates these anti-inflammatory effects by suppressing the activation of p38 and NF-κB.
